# Draft genome sequences of 12 *Escherichia coli* co-isolated with *Enterococcus* spp. from dogs with polybacterial bacteriuria at a veterinary hospital

**DOI:** 10.1128/MRA.00262-23

**Published:** 2023-08-08

**Authors:** Grayson K. Walker, Lyndy Harden, M. Mitsu Suyemoto, Siddhartha Thakur, Megan Jacob, Luke B. Borst

**Affiliations:** 1 Department of Population Health and Pathobiology, College of Veterinary Medicine, North Carolina State University, Raleigh, North Carolina, USA; Loyola University Chicago, Chicago, Illinois, USA

**Keywords:** *E. coli*, UPEC, canine urinary tract infection, polymicrobial infections

## Abstract

*Escherichia coli* are frequently co-isolated with *Enterococcus* spp. from urine cultures of dogs with urinary tract infections (UTIs). Uropathogenic *E. coli* (UPEC) are augmented by *Enterococcus* in polymicrobial UTIs. We report the draft genome sequences of 12 UPEC co-isolated with *Enterococcus* spp. from canine urinary tract infections.

## ANNOUNCEMENT

*Escherichia coli* are the most common organisms isolated from urine cultures of dogs with bacteriuria, and uropathogenic *E. coli* (UPEC) are implicated in cases of *E. coli* bacteriuria that are concurrent with clinical signs of urinary tract infections (UTIs) (1). Interestingly, UPEC are commonly co-isolated with *Enterococcus* spp. in dogs with polymicrobial bacteriuria (2). We have found that dogs with polymicrobial bacteriuria consisting of *E. coli* and *Enterococcus* spp. had worse clinical outcomes compared to dogs with single-species bacteriuria (3). Furthermore, dogs are a reservoir for these uropathogens that have the potential to be zoonotically transmitted (4). Published genomes of *E. coli* isolated from diagnosed polymicrobial UTIs are limited despite their clinical and epidemiological significance.

Twelve *E. coli* isolates from urine cultures of dogs diagnosed with polymicrobial *E. coli* and enterococcal UTIs were sequenced. All *E. coli* strains were obtained from diagnostic aerobic cultures of aseptically collected urine samples on trypticase soy agar with 5% sheep blood that contained mixed growth of only *E. coli* and *Enterococcus* from 2016 to 2020 at the NC State University Veterinary Hospital in Raleigh, NC (3). Sequenced *E. coli* and the co-isolated *Enterococcus* strains were confirmed to the species level with matrix-assisted laser desorption/ionization time of flight (MALDI-TOF) mass spectrometry using standard methods (3). Institutional ethics approvals were not required for this work.

*E. coli* genomic DNA was isolated from overnight cultures grown on trypticase soy agar with 5% sheep blood. The QIAGEN DNeasy PowerLyzer Microbial Kit (QIAGEN, Germantown, MD, USA) was used for DNA isolation. A Qubit 4.0 Fluorometer and the dsDNA high-sensitivity assay kit (ThermoFisher Scientific, Waltham, MA, USA) were utilized to assess DNA quality and quantity. The Nextera DNA Flex Library Prep Kit (Illumina, San Diego, CA, USA) was used to prepare pooled libraries with an insert size of ~350 bp. Sequencing was completed on the Illumina MiSeq platform using a MiSeq v2 Reagent Kit (500 cycles) with paired-end 150-nucleotide reads and V2 chemistry according to the manufacturer’s instructions. MicroRunQC version 3 workflow within GalaxyTrakr (5) was used for quality assessment of the sequencing reads. Contigs with a minimum length of 200 bp were assembled with the *De Novo* Assembly tool in CLC Genomics Workbench version 22.0.1 (QIAGEN, Germantown, MD, USA) using default parameters. Sequences were then annotated using the National Center for Biotechnology Information (NCBI) Prokaryotic Genome Annotation Pipeline (PGAP) version 6.3 (6). *E. coli* genome coverage ranged from 69 to 220.1× (average: 118.8×), and contig N_50_ values ranged from 57,645 to 392,281 bp (average: 229,541 bp). Assembled genomes were uploaded to the Center for Genomic Epidemiology virulence finder database version 2.0.3 (7 - 9) to detect the UPEC-associated *fimH* gene (10) in all strains using a 95% identity threshold and a minimum gene coverage of 60%. Subsequent *fimH* typing was completed using FimTyper for *E. coli* version 1.0 (11). Unless otherwise stated, default parameters were used for all sequence processing and analysis software. Isolate details, sequencing and assembly statistics, and GenBank accession numbers for raw reads and draft genome assemblies are presented in Table 1.

**TABLE 1:** NCBI accession numbers and assembly metrics of *Escherichia coli* draft genomes that were co-isolated with *Enterococcus* spp. from urine samples of dogs diagnosed with urinary tract infections from 2016 to 2020 at the NC State University Veterinary Hospital in Raleigh, NC

Strain	Co-isolated *Enterococcus* sp.^*a*^	Alias	SRA accession	No. of reads	No. of contigs^*b*^	Genome size (bp)^*c*^	G+C content (%)^*d*^	N_50_ contig size (bp)^*c*^	Genome coverage (×)^*c*^	Coding sequences^*c*^	FimH type^*e*^	Genome accession
LB-5338	*faecium*	ASM1895679v2	SRS9231170	983,691	191	4,659,864	50.8	57,645	69	4,406	H41	ABAXIT000000000
LB-5340	*faecium*	NCSU_UP63_1	SRS9231171	1,002,687	195	5,141,438	50.4	278,876	77.1	4,821	H181	ABAXHP000000000
LB-5344	*faecalis*	NCSU_UP64_1	SRS9231174	1,320,046	225	5,422,045	50.4	311,068	98.4	5,127	H9-like	ABAXJM000000000
LB-5571	*faecalis*	ASM1896157v2	SRS9231175	1,897,633	86	5,114,272	50.5	298,078	147.5	4,697	H13	ABAXNJ000000000
LB-5581	*faecium*	ASM1895767v2	SRS9231177	1,145,220	87	5,040,755	50.4	308,710	90.4	4,604	H2	ABAXJK000000000
LB-5583^*f*^	*faecalis*	NCSU_UP67_1	SRS9231176	1,156,419	229	4,911,835	50.8	392,281	94.7	4,594	H149	ABAXIU000000000
LB-5591^*f*^	*faecalis*	ASM1895609v2	SRS9231178	1,082,005	56	4,870,113	50.8	236,702	89.4	4,504	H149	ABAXHS000000000
LB-5658	*hirae*	NCSU_UP69_1	SRS9231179	962,369	228	4,790,828	50.7	131,855	82.7	4,534	H54	ABAXNM000000000
LB-5660	*gallinarum*	ASM1896163v2	SRS9231180	1,396,785	97	4,989,605	50.6	115,028	109.6	4,638	H24	ABAXNK000000000
LB-5663	*faecium*	ASM1896165v2	SRS9231181	2,851,113	74	5,128,273	50.6	205,238	220.1	4,742	H30	ABAXNL000000000
LB-5667	*faecium*	ASM1895755v2	SRS9231172	2,254,720	89	5,036,062	50.6	136,140	175.2	4,661	H38	ABAXJJ000000000
LB-5685	*faecium*	NCSU_UP73_1	SRS9231173	2,271,012	318	5,341,623	50.6	282,874	171.1	5,116	H41	ABAXJL000000000

^*a*^ *Enterococcus* was the only other bacterial species co-isolated with sequenced *E. coli* strains and was identified at the species level with MALDI-TOF mass spectrometry using standard methods (3).

^*b*^ Contigs were split into scaffolds prior to annotation.

^*c*^ Determined by the National Center for Biotechnology Information (NCBI) Prokaryotic Genome Annotation Pipeline (PGAP) (6).

^*d*^ Determined by CLC Genomics (QIAGEN, Germantown, MD, USA).

^*e*^ Determined by FimTyper for *E. coli* version 1.0 (11) after uploading draft genome assemblies to the Center for Genomic Epidemiology.

^*f*^ *E. coli* strains were isolated from the same patient diagnosed with separate urinary tract infections two calendar months apart.

## Data Availability

The draft genome assemblies have been deposited in GenBank under BioProject accession number PRJNA293225. The versions described in this announcement are the second versions. Raw read files and genome assemblies can be accessed with the Sequence Read Archive (SRA) and genome accession numbers, respectively, in Table 1.
